# Myocardial infarction and pulmonary embolism in pancreatic cancer: a case report of two manifestations of Trousseau's syndrome

**DOI:** 10.3389/fcvm.2025.1558848

**Published:** 2025-03-31

**Authors:** Seok Oh, Ju Han Kim, Doo Sun Sim, Young Joon Hong, Youngkeun Ahn, Myung Ho Jeong

**Affiliations:** ^1^ Department of Cardiology, Chonnam National University Hospital, Gwangju, Republic of Korea; ^2^ Department of Cardiology, Chonnam National University Medical School, Gwangju, Republic of Korea; ^3^ Cardiovascular Center, Gwangju Veterans Hospital, Gwangju, Republic of Korea

**Keywords:** cancer, hypercoagulability, myocardial infarction, pancreatic neoplasm, pulmonary embolism, thrombosis, venous thromboembolism

## Abstract

**Introduction:**

The simultaneous occurrence of acute myocardial infarction (AMI) and venous thromboembolism (VTE) is rare and often associated with underlying malignancies. This study reports a rare case of concurrent AMI and pulmonary thromboembolism in a patient diagnosed with pancreatic cancer.

**Case presentation:**

A 70-year-old woman presented with acute chest pain and ST-segment elevation myocardial infarction, prompting immediate percutaneous coronary intervention (PCI) with the deployment of a drug-eluting stent. Following PCI, she was treated with optimal medical therapy, including dual antiplatelet therapy. Subsequent investigations revealed pulmonary embolism, deep vein thrombosis, and imaging findings suggestive of pancreatic cancer. Anticoagulation therapy was initiated to manage the VTE. Approximately 1 month after PCI, antithrombotic agents were temporarily discontinued for pancreatic mass biopsy, confirming pancreatic ductal adenocarcinoma. The patient was referred for palliative chemotherapy.

**Discussion:**

This case highlights the clinical manifestation of Trousseau's syndrome, characterized by cancer-associated thromboembolism, and underscores the importance of coordinated antithrombotic management in complex clinical settings.

## 1 Introduction

Acute myocardial infarction (AMI) and pulmonary thromboembolism (PTE) are life-threatening conditions that may present with acute chest pain. Due to their differing pathophysiological mechanisms, the concurrent diagnosis of these two conditions is exceedingly rare.

Cancer-associated thromboembolism (CAT) is a significant cause of morbidity and mortality in cancer patients. While enhanced hypercoagulability is a well-recognized risk factor for venous thromboembolism (VTE) in this population, the link between cancer and arterial thromboembolism (ATE) remains less well understood. This study reports a rare case of concurrent AMI and PTE in a patient subsequently diagnosed with primary pancreatic cancer. This case illustrates the critical need for prompt diagnosis and intervention, including emergency percutaneous coronary intervention (PCI) to treat AMI and anticoagulation therapy to manage PTE.

## 2 Case description

A 70-year-old female patient presented to Chonnam National University Hospital with persistent and worsening chest pain. Her vital signs were as follows: blood pressure, 140/90 mmHg; pulse rate, 60 beats per minute; and oxygen saturation (measured via finger oximetry), approximately 95%. On physical examination, her heart and lung auscultations were normal, and no other abnormalities suggestive of heart failure, such as peripheral edema, were observed. A 12-lead electrocardiogram showed ST-segment elevation in the precordial leads V2-V4, along with reciprocal ST-segment depression in the inferior leads, findings consistent with an anterior ST-segment elevation myocardial infarction (Supplementary Figure S1). Laboratory tests revealed elevated levels of cardiac biomarkers, including cardiac troponin-I at 0.501 ng/ml (reference range, 0–0.05 ng/ml) and creatine kinase-MB at 3.03 ng/ml (reference range, 0–5 ng/ml). Other findings included N-terminal pro-B-type natriuretic peptide at 241 pg/ml (reference range, 0–261 pg/ml), high-sensitivity C-reactive protein at 5.05 mg/dl (reference range, 0–0.3 mg/dl), and D-dimer at 19.51 ng/ml (reference range, 0–0.55 ng/ml). Bedside transthoracic echocardiography revealed segmental akinesis of the left anterior ventricular wall and reduced left ventricular systolic function.

**Figure 1:**
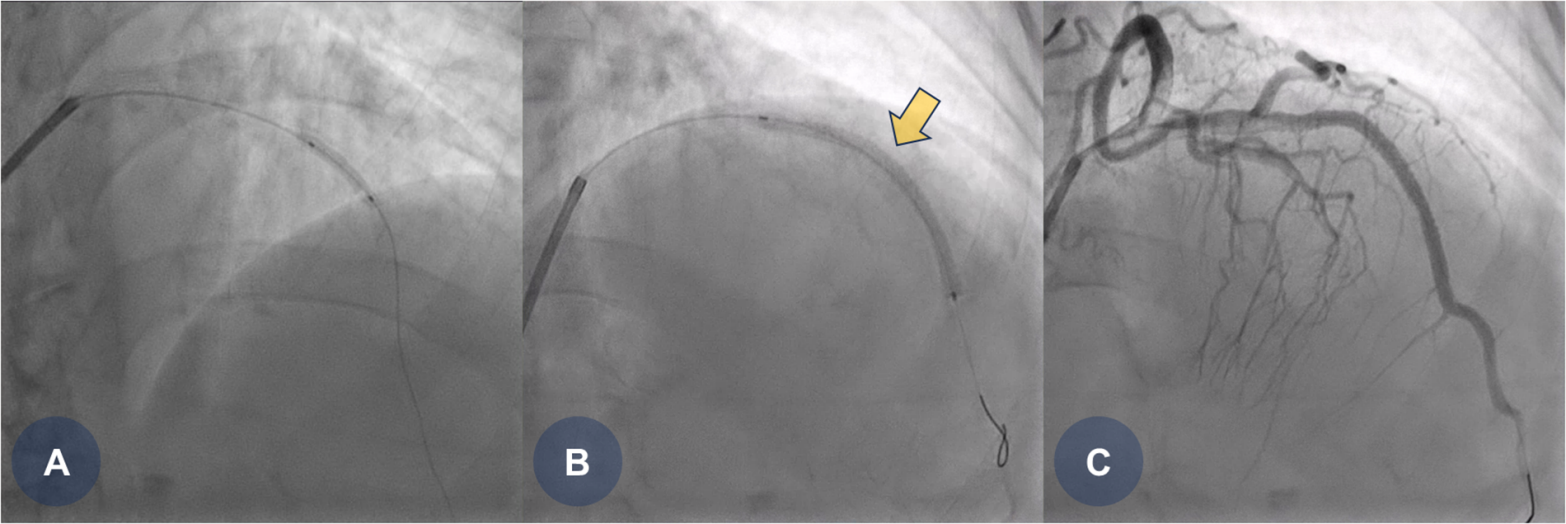
CAG findings during primary PCI. **(A)** Coronary angiography reveals total occlusion in the middle portion of the left anterior descending (LAD) artery, followed by pre-dilation ballooning using a 2.5 × 15 mm balloon catheter. **(B)** A durable-polymer everolimus-eluting stent (3.0 × 48 mm, XIENCE Skypoint; Abbott) is deployed at the middle portion of the LAD. **(C)** Final coronary angiography shows good stent expansion with no residual angiographic stenosis. CAG, coronary angiogram; LAD, left anterior descending coronary artery; PCI, percutaneous coronary intervention.

The patient was clinically suspected of having an ST-segment elevation myocardial infarction, prompting the immediate activation of the catheterization laboratory for primary PCI. Coronary angiography revealed a total occlusion in the mid-portion of the left anterior descending coronary artery. Pre-dilation ballooning was performed using a balloon catheter (Figure 1A), followed by the deployment of a durable-polymer everolimus-eluting stent (DP-EES; 3.0 × 48 mm, XIENCE™ Skypoint™; Abbott) at the site of the obstruction (Figures 1B,C, yellow arrow). Following successful PCI, the patient was admitted to the general ward for post-PCI management. She was treated with optimal medical therapy, including dual antiplatelet therapy (DAPT), beta-blockers, angiotensin receptor blockers, and high-intensity statins.

**Figure 2:**
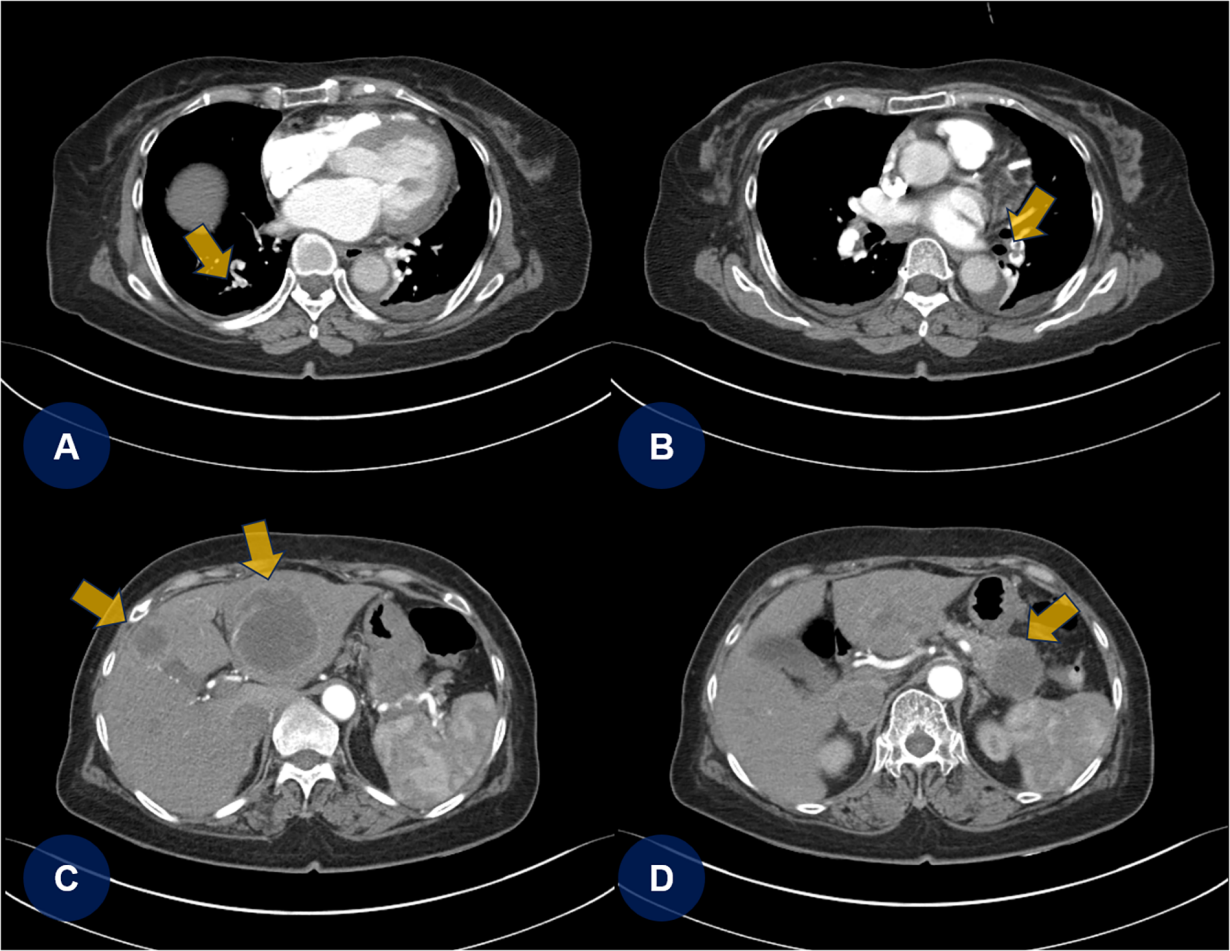
Chest and abdominal CTA. **(A,B)** Chest CTA reveals multifocal pulmonary thromboembolisms (yellow arrows). **(C,D)** Abdominal CTA shows multifocal round-shaped hepatic lesions with a solitary mass-like lesion in the tail of the pancreas. CTA, computed tomography angiography; PTE, pulmonary thromboembolism.

**Figure 3:**
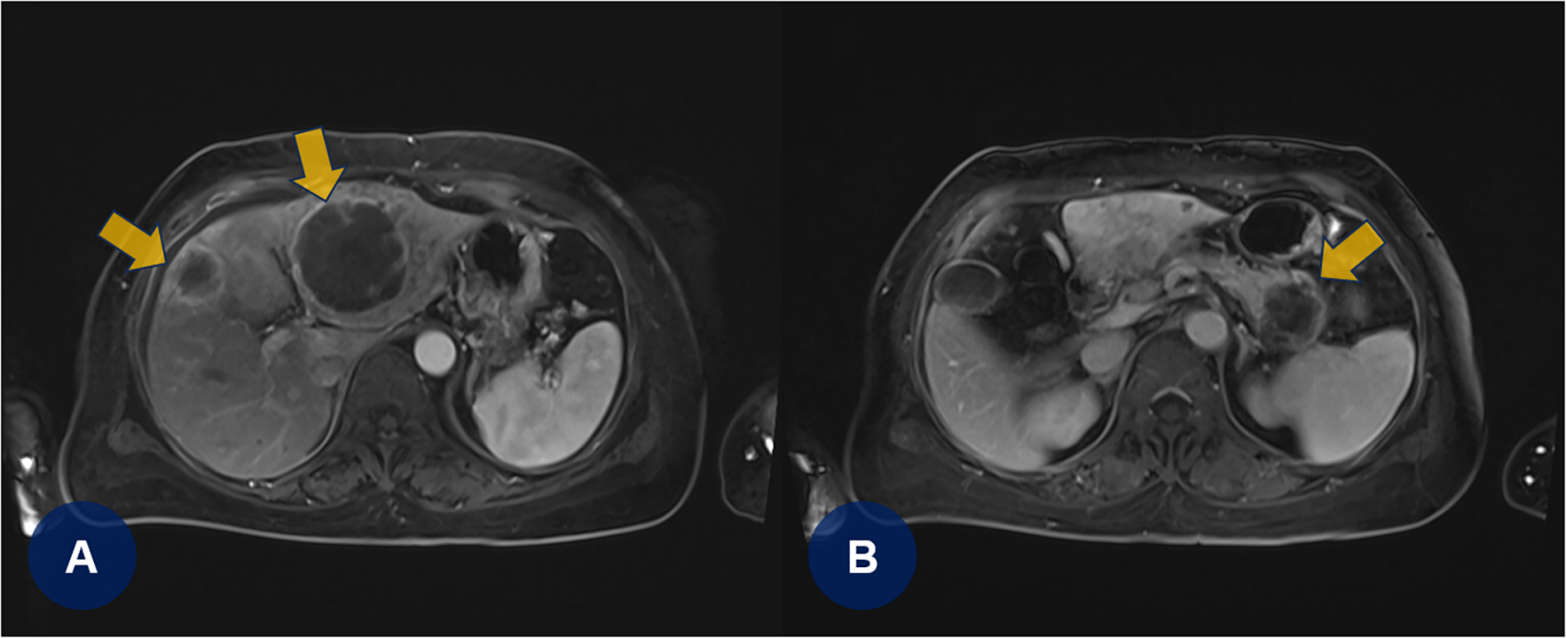
(**A,B**) Dynamic contrast-enhanced magnetic resonance imaging confirms primary pancreatic cancer with hepatic metastases (yellowish arrows).

A few days later, the patient experienced recurrent abdominal discomfort accompanied by intermittent episodes of mild fever. SARS-CoV-2 and influenza rapid antigen test results were negative. The treating physician decided to perform chest and abdominal computed tomography angiography (CTA) to identify potential underlying abnormalities, including inflammation, infection, thromboembolic lesions, or malignancies. CTA revealed PTEs (Figures 2A,B, yellow arrows) and multifocal round hepatic lesions along with a solitary mass-like lesion in the pancreatic tail (Figures 2C,D, yellow arrows), suggestive of primary pancreatic cancer. Additional venous CTA confirmed the presence of deep vein thrombosis (DVT) in both popliteal veins (Supplementary Figure S2). To manage the PTE and DVT, intravenous heparin was initiated. One week later, the heparin therapy was transitioned to a direct oral anticoagulant (DOAC), apixaban (5 mg twice daily). Dynamic contrast-enhanced magnetic resonance imaging subsequently confirmed the diagnosis of primary pancreatic cancer with hepatic metastasis (Figures 3A,B, yellow arrows).

To address the pancreatic cancer diagnosis, the treating physician consulted the gastroenterology department, which performed an endoscopic ultrasound-guided fine-needle aspiration (EUS-FNA) of the pancreatic mass. Considering the recent PCI performed for AMI, the multidisciplinary team recognized that interrupting DAPT posed a significant risk of ischemia. However, to avoid delaying the histopathological diagnosis of pancreatic cancer, the team opted for an alternative approach. They scheduled the biopsy for 1 month after PCI, temporarily discontinuing DAPT during this period, despite the associated risk of stent thrombosis.

**Figure 4:**
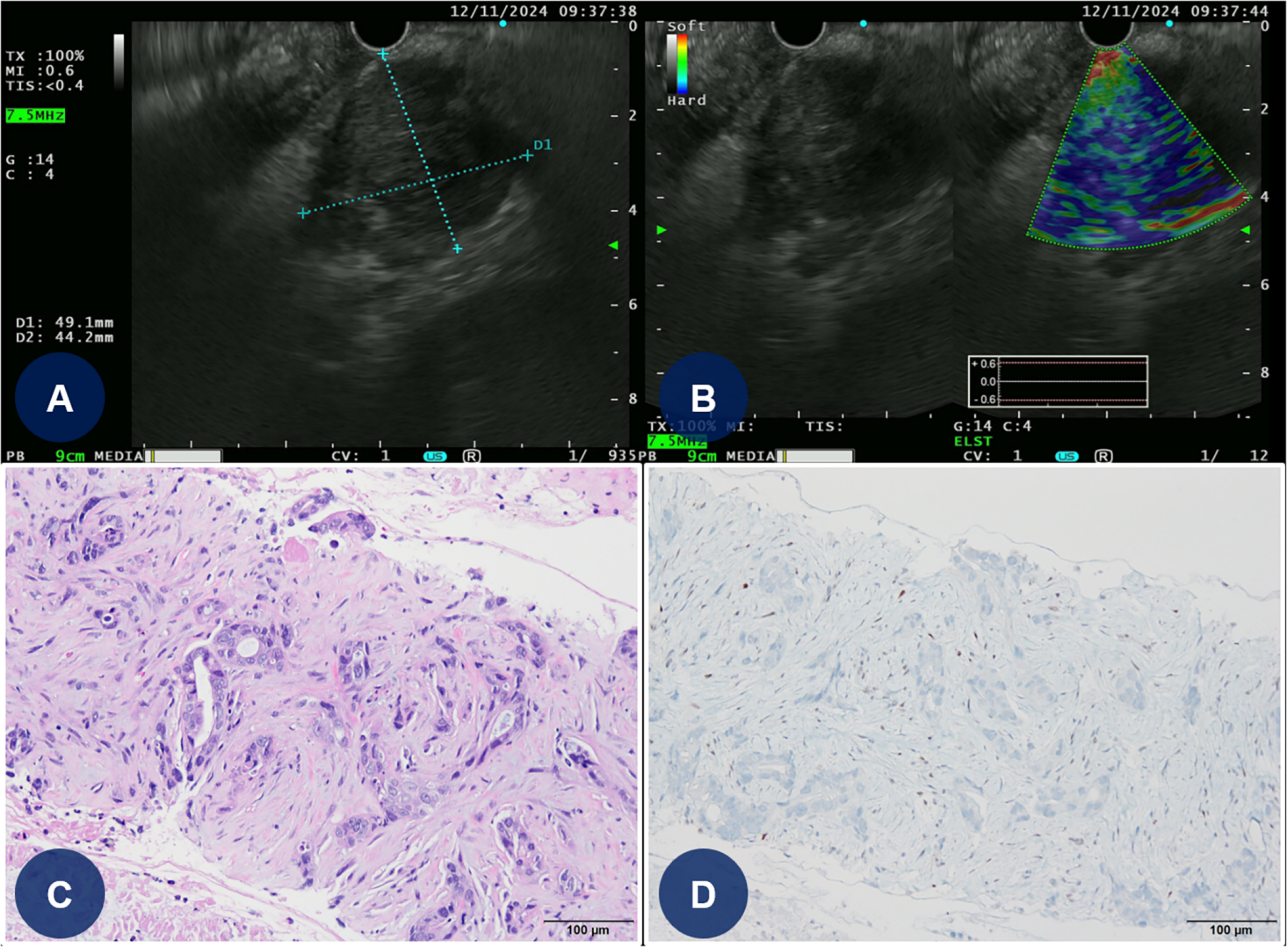
EUS-guided FNA and histopathologic findings of the pancreatic mass. **(A,B)** EUS-guided FNA of the pancreatic mass **(C)** Light microscopic finding reveals angulated glandular structures with prominent nuclear atypia and extensive stromal desmoplastic reaction (hematoxylin and eosin-stained slide at 200× magnification). **(D)** Immunohistochemical strain for p53 shows aberrant expression pattern (original magnification at 200× magnification). EUS, endoscopic ultrasound; FNA, fine-needle aspiration.

Approximately 1 month after PCI, the patient temporarily discontinued DAPT for 5 days and DOAC therapy for 2 days to undergo EUS-FNA (Figures 4A,B). Following the biopsy procedure for pancreatic cancer, all antithrombotic therapies (DAPT and DOAC) were promptly resumed. Pathological analysis confirmed the diagnosis of a moderately differentiated pancreatic ductal adenocarcinoma with a p53 mutation (Figures 4C,D). The patient was subsequently discharged from the hospital and transferred to another medical center to begin palliative chemotherapy for pancreatic cancer.

## 3 Discussion

**Figure 5:**
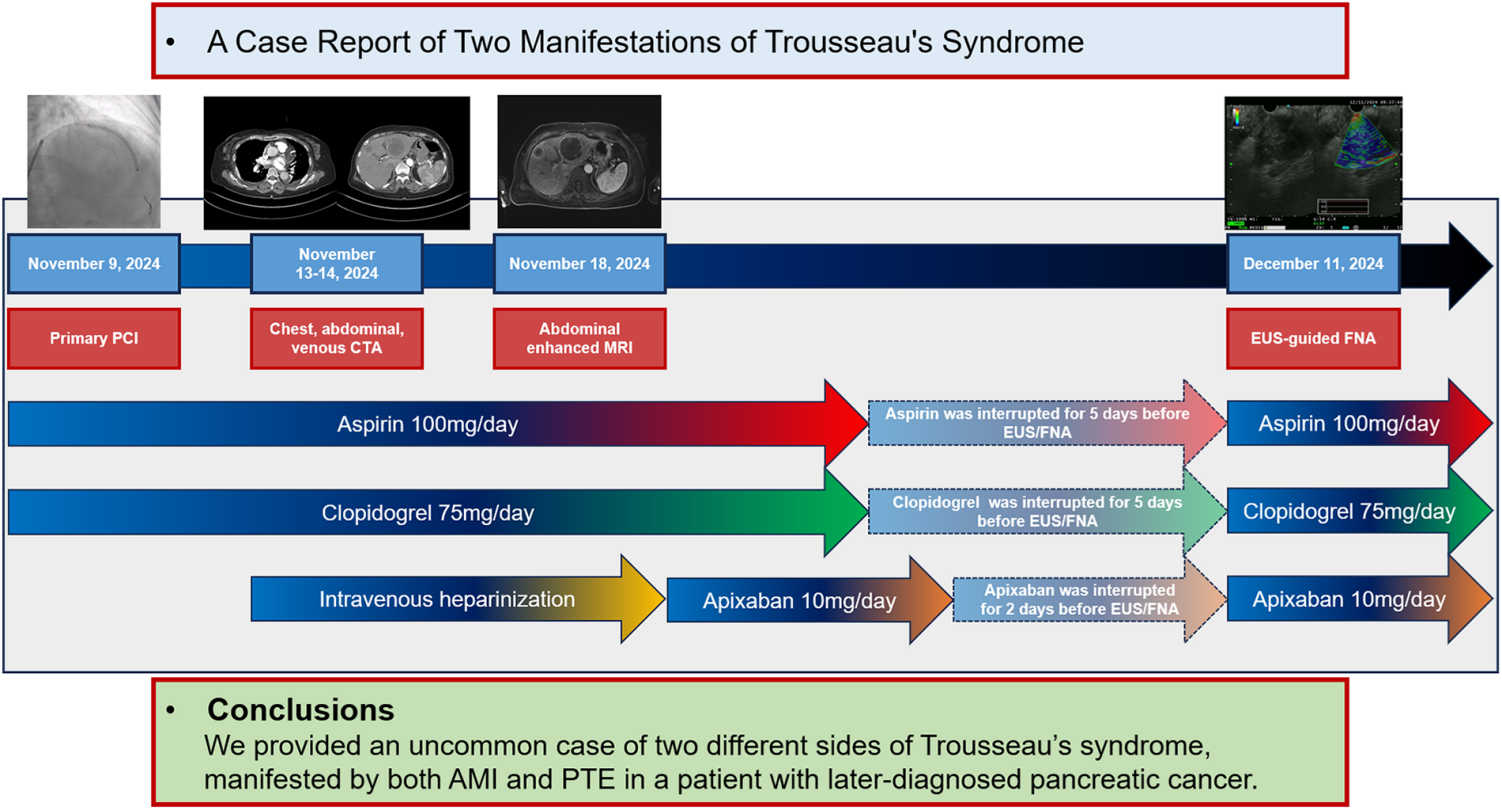
Timeline of the clinical case. AMI, acute myocardial infarction; CTA, computed tomography angiography; EUS, endoscopic ultrasound; FNA, fine-needle aspiration; MRI, magnetic resonance imaging; PCI, percutaneous coronary intervention; PTE, pulmonary thromboembolism.

This case highlights the simultaneous occurrence of AMI and PTE in a patient with underlying pancreatic cancer. The patient underwent successful PCI and received optimal medical treatment. Despite the substantial ischemic risk, the medical team proceeded with an “off-label” interruption of antithrombotic therapy to facilitate a timely biopsy, ensuring a prompt diagnosis and initiation of treatment for pancreatic cancer (Figure 5).

CAT, also known as Trousseau's syndrome, is widely recognized as a major health issue. Armand Trousseau was the first to investigate the link between cancer and an increased risk of thrombosis (1). Blood hypercoagulability can increase the risk of thromboembolic events in patients with cancer, which in turn increases the incidence of ischemic cardiovascular events (2–4). While VTE, including PTE and DVT, is the most common manifestation of CAT and an important cause of mortality in this population, the proposed association has primarily focused on VTE (4). The epidemiology of ATE, such as AMI or ischemic stroke, has received less attention. Although several clinical studies have shown a significant association between certain types of cancer and ATE (5–8), the incidence of ATE appears to be lower than that of VTE (6, 7). However, a large-scale randomized controlled trial reported that the incidence of ATE at 6 months after lung cancer diagnosis was 8.3%, with AMI accounting for 2.0% (8). Furthermore, a nationwide observational study from Korea demonstrated that patients with newly diagnosed cancer had an increased risk of ATE (9).

A review of the literature reveals several reports of concomitant AMI and PTE as the first clinical presentation of malignancy (10–12). Similar to these cases, our patient exhibited both AMI and PTE before being diagnosed with primary pancreatic cancer. Such occurrences are extremely rare and require the use of both antiplatelet therapies and anticoagulants to prevent further thromboembolic events (12). However, many clinicians may face clinical situations where temporary interruption of these antithrombotic treatments is necessary. In our case, as the patient had undergone PCI with a DP-EES approximately 1 month before the EUS-FNA, the risk of stent thrombosis following the interruption of both DAPT and DOAC treatment was extremely high, despite the elevated bleeding risk associated with EUS-FNA (13, 14).

Most international guidelines recommend DAPT for 1 year in acute coronary syndrome, including AMI (15). However, despite ongoing debate, there is increasing evidence supporting a shorter duration of DAPT followed by single antiplatelet therapy to balance ischemic and bleeding risks (16–20). As a result, the 2023 European Society of Cardiology guidelines suggest that a reduction in DAPT treatment time followed by aspirin or a P2Y12 inhibitor monotherapy may be considered for patients with a high bleeding risk (HBR) (15). Moreover, the DP-EES (XIENCE™ Skypoint™; Abbott) used in our case has demonstrated non-inferiority for 1- or 3-month DAPT compared with that for 6- or 12-month DAPT among HBR patients (21). This stent has also received the Conformité Européenne mark in Europe for shortened durations of DAPT in selected patients.

Our case highlights some important clinical points. First, when ATE and VTE occur simultaneously, very-high-intensity antithrombotic treatment using both antiplatelet therapies and anticoagulants is required. However, as mentioned earlier, in cases with a HBR, interruption of the antithrombotic treatment may be clinically inevitable. Particularly, when primary PCI has recently been performed and both bleeding and ischemic risks are extremely high, careful consideration is needed to weigh the risk vs. benefit of interrupting the antithrombotic treatment. Second, as metallic drug-eluting stents (DESs) have revolutionized PCI with marked technologic refinements in terms of metallic platform, polymer, and antiproliferative drugs, the rate of stent thrombosis has been dramatically reduced (22–25). In other words, the advent of newer-generation DESs with thinner struts and enhanced polymer biocompatibility has significantly contributed to a reduction in post-PCI ischemic events (23). In light of these advancements, international guidelines, considering the totality of evidence from the scientific literature, have endorsed alternative DAPT strategies, such as DAPT de-escalation or treatment length reduction to suit individual patient profiles (15). As previously mentioned, we deployed a new-generation DES (XIENCE™ Skypoint™; Abbott) supported by clinical evidence for the shortened durations of DAPT.

In conclusion, we present a rare case of Trousseau's syndrome manifesting in two distinct forms, AMI and PTE, in a patient eventually diagnosed with pancreatic cancer. Both these conditions are major causes of mortality and require prompt medical and interventional treatment.

## Data Availability

The raw data supporting the conclusions of this article will be made available by the authors, without undue reservation.

## Supplementary material

Supplementary Figure 1 Initial 12-lead electrocardiogram.

Supplementary Figure 2 Additional venous CTA confirms DVT in both popliteal veins (yellow arrows). CTA, computed tomography angiography; DVT, deep vein thrombosis.
