# Supplementary figures and images for: Myocardial infarction and pulmonary embolism in pancreatic cancer: a case report of two manifestations of Trousseau's syndrome
Source: Front Cardiovasc Med. 2025 Mar 31;12:1558848. doi: 10.3389/fcvm.2025.1558848 (PMC11994600; doi:10.3389/fcvm.2025.1558848)

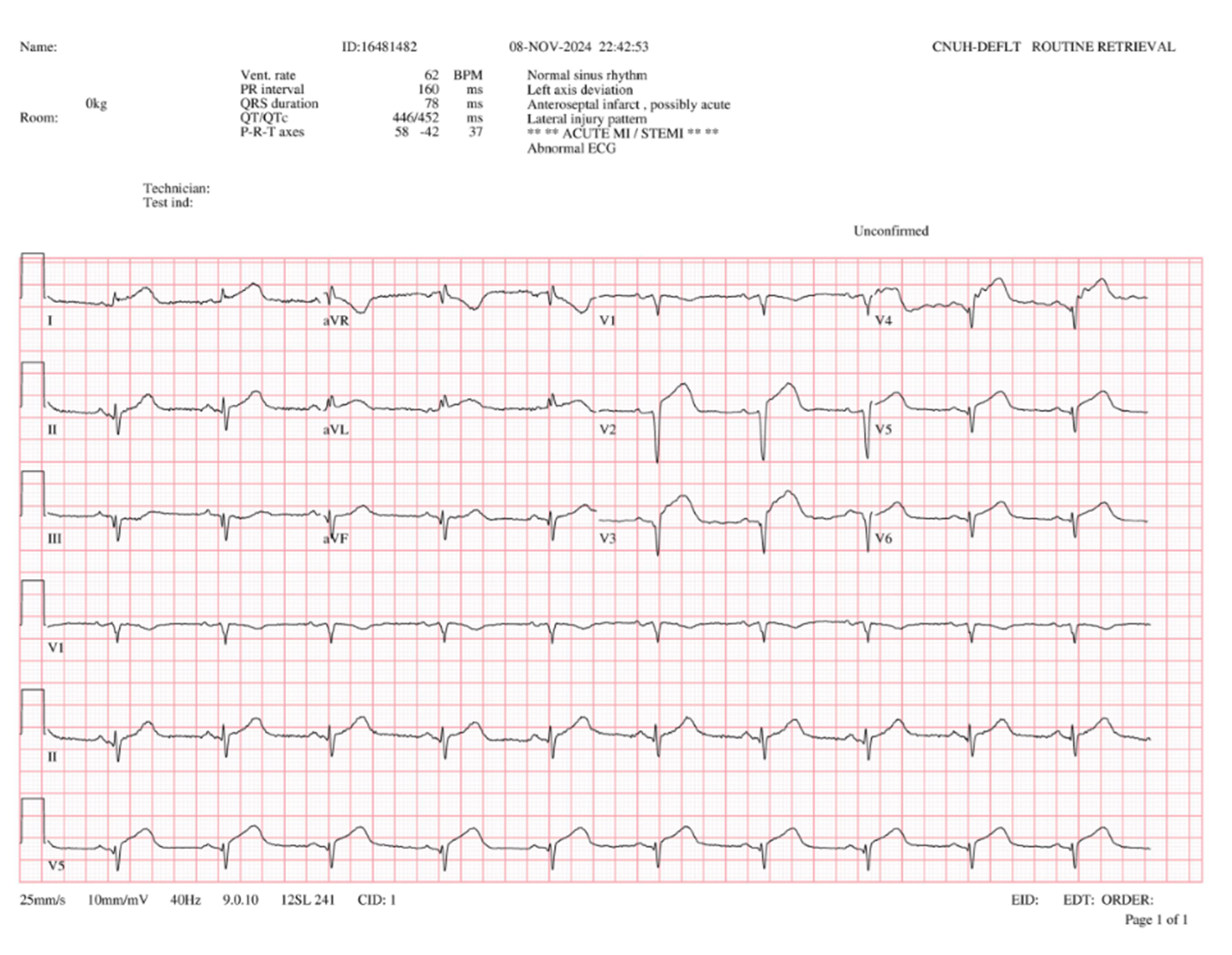

Supplement: Supplementary Figure 1 — Initial 12-lead electrocardiogram. [file Image1.tif]

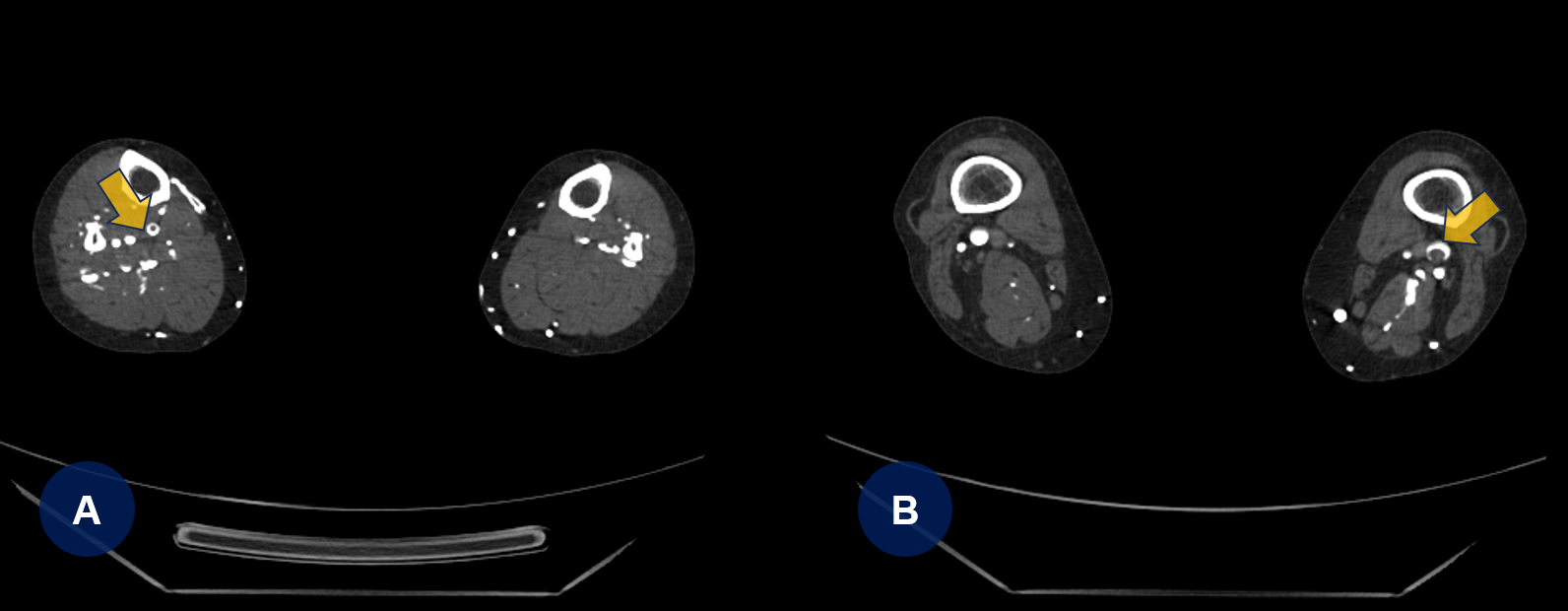

Supplement: Supplementary Figure 2 — Additional venous CTA confirms DVT in both popliteal veins (yellow arrows). CTA, computed tomography angiography; DVT, deep vein thrombosis. [file Image2.tif]

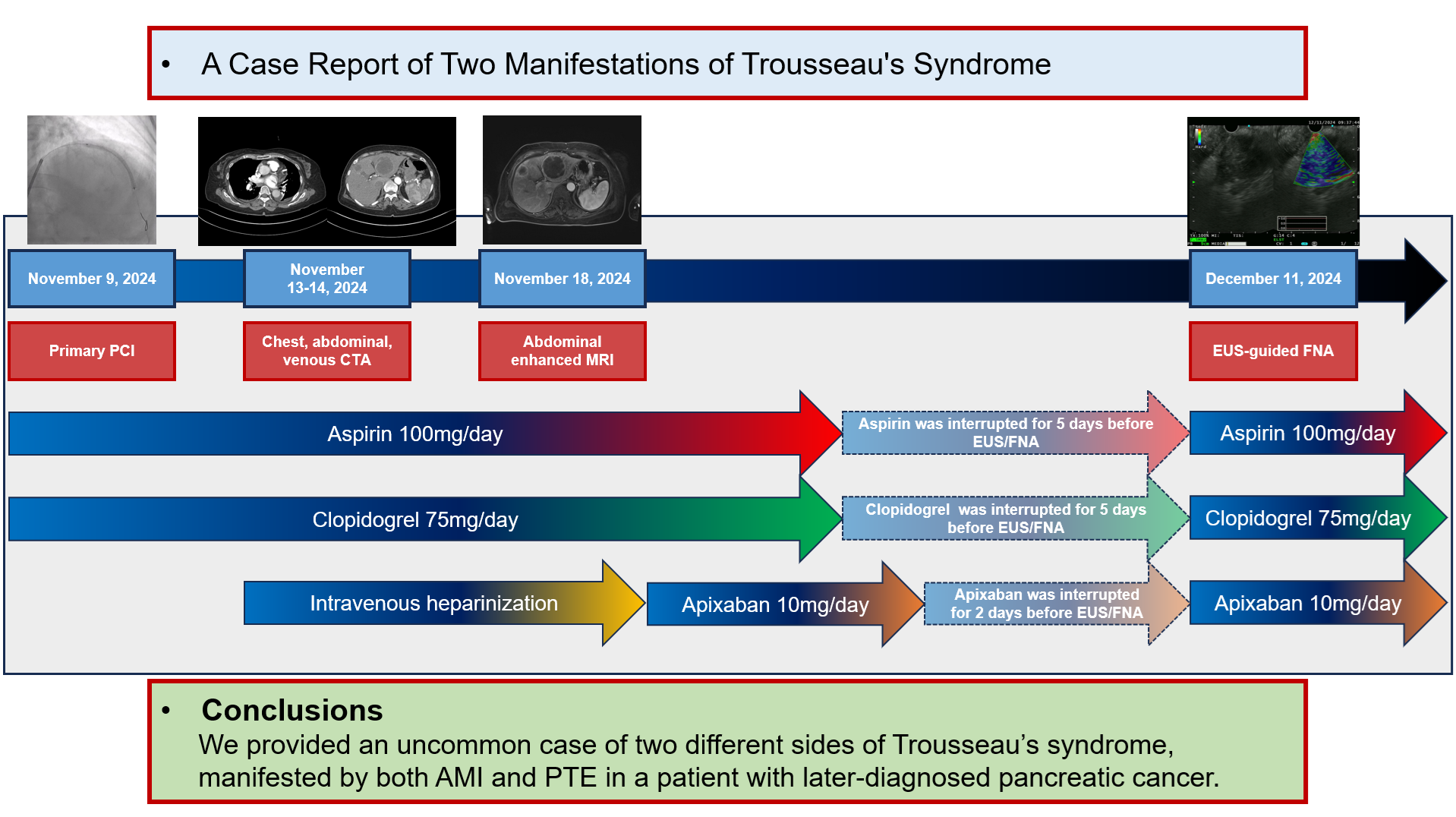

Supplement: Supplementary file 3 [file Image3.tif]
